# Barriers to exchanging healthcare information in inter-municipal healthcare services: a qualitative case study

**DOI:** 10.1186/s12911-018-0701-z

**Published:** 2018-11-07

**Authors:** Elisabeth Holen-Rabbersvik, Elin Thygesen, Tom Roar Eikebrokk, Rune Werner Fensli, Åshild Slettebø

**Affiliations:** 1 Department of Health and Nursing Science, Centre for eHealth, University of Agder, PO Box 509, 4898 Grimstad, Norway; 2 Department of Information Systems, University of Agder, PO Box 422, 4604 Kristiansand, Norway; 3 Department of Information and Communication Technology, Centre for eHealth, University of Agder, PO Box 509, 4898 Grimstad, Norway; 4 Department of Health and Nursing Science, Centre for care research, South University of Agder, PO Box 509, 4898 Grimstad, Norway

**Keywords:** Inter-municipal cooperation, Case study, Information sharing, Information infrastructure, Inter-organisational cooperation

## Abstract

**Background:**

In recent years, inter-municipal cooperation in healthcare services has been an important measure implemented to meet future demographic changes in western countries. This entails an increased focus on communication and information sharing across organisational borders. Technology enables efficient and effective solutions to enhance such cooperation. However, the systems in the healthcare sector tend not to communicate with one another. There is a lack of literature focusing on communication and information sharing in inter-municipal healthcare services. The aim of this article is to investigate both the characteristics of communication and information sharing, and the factors that serve as barriers to communication and information sharing for employees in inter-municipal healthcare services.

**Methods:**

In this study, a qualitative case study approach is used to investigate both characteristics of communication and information sharing, and factors enabling barriers to communication and information sharing for employees in newly established inter-municipal healthcare services. Data collection methods were individual interviews, focus group interviews, observation studies and a workshop. A total of 18 persons participated in the study. The interviews, observations and workshop were conducted over a period of ten months.

**Results:**

Communication and information sharing practices were found to be complex and characterised by multiple actors, information types and a combination of multiple actions. Findings indicate that 1. IT capability and usability 2. Differences 3. Privacy, confidentiality and security and 4. Awareness are all factors enabling barriers to communication and information sharing in inter-municipal healthcare services. Specifically, these barriers were related to lack of EHR usability, inadequate workflow processes, digital systems incompatibility, the understanding of needs in different systems and knowledge and practices regarding privacy and confidentiality.

**Conclusion:**

By focusing on the context of inter-municipal cooperation when assessing communication and information sharing in healthcare services, this article contributes to close a gap in existing knowledge. The perspective of the employees provides useful insight, and findings can be relevant for future theory development and for managers and policymakers in inter-municipal services.

## Background

Because of future demographic changes, including an increasing number of older people in western countries, there is a need to enhance the efficiency of healthcare services. Healthcare reforms often emphasise coordination in both the design and delivery of healthcare services. Both intra- and inter organisational, vertical and horizontal coordination, have become objects of increased focus in the field of health services research [1, 2], for example in coordination between primary healthcare and specialized health care, and within various providers of primary healthcare. To arrange new and specialized services in health- and social services, several inter-municipal cooperations (IMC), have been established in recent years [3]. This entails an increased focus on communication and information sharing across municipal borders. Technology opens for several methods of communication and information sharing between collaborative partners and provides the ability to enhance the effectiveness and efficiency of inter-organisational cooperation. Information systems play a key role in supporting healthcare professionals and the delivery of healthcare services. In the public sector, cross boundary information is increasingly important [4]. Inter-municipal healthcare service production implies a large amount of horizontal information sharing across the involved municipalities. Horizontal information sharing is normally found to be more difficult than vertical information sharing [5].

Governments express the need for new and improved ways to deliver healthcare services and expects changes to be implemented in the sector. This implies an assumption on the part of the government that the infrastructure of healthcare information is currently suitable for new working practices. However, it is known that despite technological maturity among European healthcare organisations, the systems tend not to communicate with one another [6]. Information and communication technology (ICT) systems that are unable to share information with other ICT systems are a threat to optimal communication and information sharing, and consequently to IMC.

In a framework of inter-organisational information sharing in the public sector, several success factors at the practitioner’s level are identified, including minimisation of changes in existing internal processes and information flow, and a strong leadership to support information sharing efforts [5]. In newly established inter-municipal services, there is no established process and information flow, hence the framework is less applicable. Furthermore, it is well known that management of inter-municipal cooperation is challenging [3, 7]. There is a lack of literature focusing on communication and information sharing in inter-municipal healthcare services. To close the gap in existing knowledge, there is a need for a specific focus on the context of inter-municipal cooperation when assessing communication and information sharing in healthcare services.

The aim of this article is to investigate both characteristics of communication and information sharing, and factors that enable barriers to communication when employees in inter-municipal cooperation communicate and share information. Specifically, the following research questions will be explored:

RQ1: What characterises communication and information sharing practices in inter-municipal healthcare services?

RQ2: Which factors can contribute to barriers for communication and information sharing in newly established inter-municipal healthcare services?

### Related research and theory

The present study uses an inductive approach to identify and elaborate on communication and information sharing in inter-municipal healthcare services from the perspective of the employees. Communication and information sharing are essential for efficient collaboration. To answer the research questions, we apply related research that sheds light on the organisational aspects of inter-municipal cooperation, in addition to research targeting the technology related to communication and information sharing. A summary of previous research specifically targeting inter-organisational cooperation, information infrastructure, including the eHealth perspective, information sharing and the Unified Theory of Acceptance and Use of Technology (UTAUT), will be presented. The inter-disciplinary theories and research presented are not necessarily interrelated in themselves, but each concept can provide unique insight into the findings in this study. The aim is to gain a broad theoretical perspective on this inductive research.

#### Inter-organisational cooperation

Factors like devolution, technical development, scarce resources and changing demography characterises the public sector and have led to increased focus on collaboration [8]. This includes both vertical and horizontal collaboration within and across organisations and professions [9]. Organisations are abstract phenomena and may be viewed as cultural artefacts created through human interaction. Over time, the organisations tend to become “institutionalised” and their roles and tasks are accepted and institutionalised by the broader environment. Inter-organisational integration, according to institutional economic theory, can be achieved through a management hierarchy with a top-down coordination in organisations, in a market coopetition with contractual relations between organisations [10]. It can also manifest itself in the form of networks, which is collaboration between organisations without a common hierarchy [11]. Inter-municipal cooperation can be considered the network form of inter-organisational integration.

##### Network governance

In recent decades, the establishment of networks has increased. There have been many suggestions for the definition of the organisation of networks, as an alternative to hierarchical organisation [12–14]. One widely accepted definition is from Jones, Hesterly and Borgatti: “Network Governance involves a select, persistent and structured set of autonomous firms (as well as non-profit agencies) engaged in creating products or services based on implicit and open-ended contracts to adapt to environmental contingencies and to coordinate and safeguard exchanges. These contracts are socially – not legally – binding” [15]. The term “governance” is used instead of government, as it captures the process and approach to the organising of networks referred to in the definition. In governance network theory, it is asserted that “the network form of governance is a response to exchange conditions of asset specificity, demand uncertainty, task complexity, and frequency” [15].

Network governance can take different forms and has been categorised in the following three types: shared governance, lead organisation and network administrative organisation. Within all three forms of organisations, three basic tensions are found to be inherent; efficiency versus inclusiveness, internal versus external, legitimacy and flexibility versus stability [16]. Both the organising of the network, as well as management of the tensions existing in the various networks are deemed important for success. Tension management is critical for network effectiveness [16]. However, there is a lack of knowledge and consensus regarding the kind of management that should be applied in this context, and the way in which it should be applied. In public sector research, network organising has been widely addressed, in terms of both network structure and context (see e.g. K Huang and KG Provan [17] and LJ O’Toole Jr. and KJ Meier [18]) as well as the management and coordination of public networks (see e.g. M Kort and EH Klijn [19] and WJ Kickert, E-H Klijn and JF Koppenjan [20]). Recently, there has been an increased focus on the importance of managers facilitating information sharing and communication in networks [21, 22], however, exactly how and what areas managers should focus on has not been fully elaborated.

#### Information infrastructure

The information infrastructure is a prerequisite for the facilitation of communication and information sharing in inter-municipal healthcare services. The term “infrastructure” refers to equipment necessary to facilitate human activities in society such as roads, railways, harbours, waste management and electricity supply. In addition, the large amount of information exchanged in society needs supportive infrastructure. Infrastructure is described as fundamentally and always a relation, never a thing [23] and is often invisible and taken for granted by employees [24]. There is a diffuse boundary between the technological and organisational means of information processing. People, routines, forms and classification systems are as integral to the information handling as computer cables and web protocols [25].

##### eHealth infrastructure

In this study, we rely upon the European Commission which refers to eHealth as “tools and services using information and communication technologies (ICTs) that can improve prevention, treatment, monitoring and management” [26]. In recent decades, there have been overarching strategies and plans focusing on digitalisation of healthcare services around the globe, but initiatives do not seem to have had the expected effect. In the eHealth Action Plan from 2004, for example, there are several measures to foster a widespread adoption of eHealth technologies around Europe [27]. However, in the latest eHealth Action Plan it is stated that the envisioned interoperable eHealth infrastructure in Europe has not been realised and the promise of eHealth “remains largely unfulfilled” [28].

There are a number of systems under the umbrella of eHealth, for example Picture Archiving and Communication Systems (PACS), Radiology Information Systems (RIS), Patient Administrative Systems (PAS) and the Electronic Health Record (EHR) [29]. The EHR plays a central role in healthcare institutions. Its primary purpose is “…the support of continuing, efficient and quality integrated healthcare…It contains information which is retrospective, concurrent and prospective” [30], Chapter 2.10]. In Europe, healthcare and eHealth, is to a large degree a public responsibility. In many countries, collaboration has been the driving force for implementation of eHealth initiatives [31]. Inter-organisational eHealth infrastructures, both horizontal and vertical, are important for a number of stakeholders such as healthcare personnel, researchers and public authorities. When enhancing eHealth infrastructure across organisational boundaries, standards regarding interoperability, terminology and nomenclature are crucial components [29].

#### Information sharing

During recent decades, there has been a shift from information protection to information sharing across organisational borders [5]. Such sharing of information is regarded as important for increasing the organisations’ efficiency and performance, for example on issues like anti-terrorism and public health [5]. When focusing on inter-organisational information sharing, also the context of intra-organisational and intra-personal information sharing are found to be interrelated [5]. Three primary factors are found to influence inter-organisational information sharing: organisational, technological, political and policy perspectives [5]. The expected benefits of starting collaborative eGovernment initiatives are affected by perceived impediments and prior experience [32]. Information sharing is found to be a challenge both in public and private organisations as it often requires collaboration between several organisations to share information and, in some cases, to integrate business processes [32]. To minimize changes in internal processes and information flow, is one suggestion to improve organisations’ chances for successful information sharing. In addition, activities like “promotion of a culture of information stewardship as opposed to ownership; strong leadership support to information sharing efforts; legislative and regulative mandates; a reward system that promotes information sharing both within and across organisations; the establishment of shared goals; and the development of ongoing trusted relationships based on mutual understanding of needs and concerns and shared responsibility…” [5] are all positive actions designed to promote inter- organisational information sharing.

##### Unified Theory of Acceptance and Use of Technology (UTAUT)

**Fig. 1:**
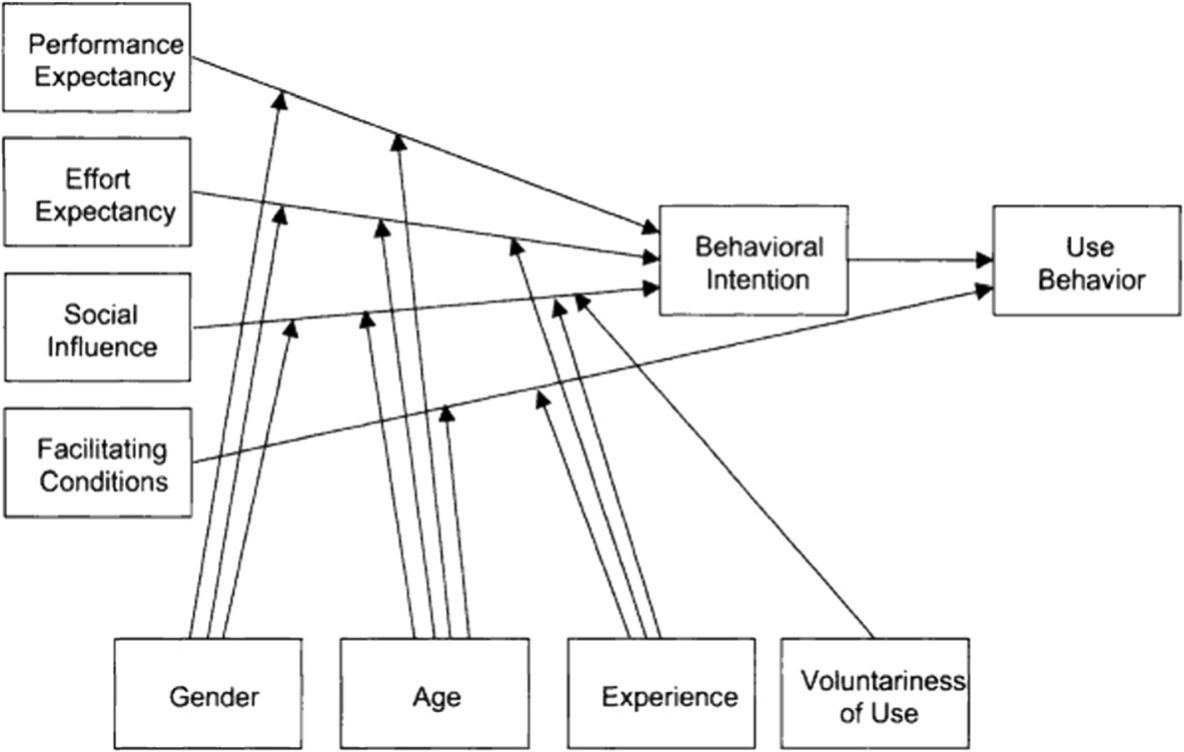
The UTAUT Model. Permission: Figure 3 from Venkatesh et al. [33]. Copyright © 2003, Regents of the University of Minnesota. Reprinted by permission

When establishing a new IMC, the use of information technology is an integrated part of the work and support communication and information sharing needs across organisational boundaries. When implementing new inter-municipal services, the technology can be new or used in a new way, relying on existing systems. Either way, employees must use and implement the information technology in a new setting. This implies that employees must accept and use the technology. In recent years, the Unified Theory of Acceptance and Use of Technology (UTAUT) [33] has been widely used by Information systems (IS)/ Information technology (IT) researchers and have been found to be useful in the context of healthcare [34, 35]. The model provides a useful tool for managers by providing an understanding for the many factors that can influence user acceptance for IS, hence the ability to design interventions targeting the end users [33]. It integrates eight theories into one model, that can provide insight into factors hindering or enabling the adoption and use of technology. Performance expectancy, Effort Expectancy and Social Influence are described as impacting Behavioural Intentioin, and hence Use Behaviour. Facilitating Conditions are described as directly impacting Use Behaviour (Fig. 1).

In this Background section, we have included related research and theories which is found to have an impact on information sharing and communication in inter-municipal healthcare services. The first section; Inter-organisational cooperation, including network governance provides insight into the organisational aspects of IMC. The second section; Information infrastructure sheds light on prerequisites for information sharing in general and in eHealth settings in particular. In public administration today, information technology is an obvious and necessary tool in everyday work. Therefore, the third section; Information sharing, deals with information sharing particularly across organisations, and include factors hindering or enabling the adoption and use of technology.

Following this Background section, methodology, including setting, design, data collection, participants, analysis and ethical considerations are presented in Methods section. Results section presents the results structured on the two research questions. In Discussion section, the results are discussed in relation to previous research along present findings that are distinctive for the context of communication and information sharing in inter-municipal healthcare services, in addition to a discussion of the implications for practice and limitations. Finally, concluding remarks are given.

## Methods

### Setting

The study was conducted in Norway, a European country with approximately 5 million inhabitants. Norway has three levels of political administration: national government, counties and municipalities. In addition, the country is divided into districts, which are larger than the municipalities and smaller than the counties. The districts are characterised by common culture, dialects or geography and may cross county borders. The Norwegian Coordination Reform [36] was implemented in 2012. The reform’s new directions increased the municipalities responsibility for health and social care. Because of diversity among municipalities in terms of population density and, as a result, access to competence, the municipalities were encouraged to collaborate on service delivery.

In this study, two formal districts are included, including councils and representatives from each participating municipality. Both districts had established various inter-municipal health initiatives to address new working practices based on the Coordination Reform [36]. District 1 had fewer than 10,000 inhabitants distributed among four municipalities, and districts 2 had some 35,000–40,000 inhabitants distributed among six municipalities.

### Design

In the present study a qualitative case study approach was used. The boundaries between the phenomena of communication and information sharing in IMC and the context are not clear, and the contextual conditions were regarded as relevant to the findings. In this situation, Yin [37] recommends that a case study approach should be considered. A descriptive approach has been applied to answer the first research question, and an exploratory approach to answer the second research question. Individual interviews, focus group interviews, observation studies and one workshop were conducted. Multiple data sources facilitate the acquisition of a holistic understanding of the phenomenon and enhance data credibility [37, 38]. The different data collection methods are used to elucidate divergent aspects of the phenomenon. The individual interviews provide an in-depth understanding of the phenomenon from the perspective of the individual; the observational studies provide a thorough knowledge of the phenomenon and guided our inclusion of relevant participants; the focus group interviews generate dialogue and subsequent reflection among the participants; and the workshop contributes toward validating our findings.

### Participants

Managers in the two districts identified persons who met the following criteria for inclusion: they were employed in an inter-municipal health- or social service delivery, either as project leaders, employees of an inter-municipal team, or as persons related to the ICT aspects of inter-municipal services. In District 1, the manager identified eight participants; the members of the dementia team, the ICT-consultant and the project leader of the inter-municipal (IM) services. In District 2, the manager identified four participants. Because of the need for an in-depth study in District 1, the collaborative partners in the various municipalities had to be contacted. During primary interviews and observation studies in District 1, the coordinator of the inter-municipal team, identified collaborative partners, and these were then contacted. Their managers approved their participation. The researcher contacted the general practitioners (GPs) directly. All participants volunteered to participate when requested to do so, except for one GP who lacked the resources to allow him to participate. As an alternative, the GP recruited his secretary who substituted for him in the interview (Table 1).

**Table 1:** Participants

	Profession	Sex	Age	Data collection methods
District 1	Psychologist	Female	20–29	• Qualitative interview
	Substance abuse therapist	Female	20–29	• Qualitative interview • Observational study • Phone interview
	ICT manager	Female	40–49	• Qualitative interview
	Dementia coordinator	Male	50–59	• Workshop • Focus group interview • Two observational studies
	Dementia contact 1	Female	50–59	• Workshop • Focus group interview
	Dementia contact 2	Female	50–59	• Workshop • Focus group interview • Observational study
	Dementia contact 3	Female	20–29	• Workshop • Focus group interview
	Dementia contact 4	Female	30–39	• Workshop • Focus group interview • Observational study
	Consulting doctor	Male	30–39	• Workshop • Focus group interview
	General practitioner	Female	30–39	• Qualitative interview
	Medical secretary	Female	?	• Qualitative interview
	Community nurse manager 1	Female	30–39	• Qualitative interview
	Community nurse manager 2	Female	20–29	• Qualitative interview
	Project manager for substance abuse therapist and psychologist	Female	50–59	• Qualitative interview
District 2	Occupational therapist	Female	30–39	• Qualitative interview
	“Palliation in Vik” project manager/coordinator	Female	30–39	• Qualitative interview
	Manager of a substance abuse team	Female	30–39	• Qualitative interview
	ICT consultant	Female	30–39	• Qualitative interview

A total of 18 persons participated in the study, including seven who participated more than once. The interviews and workshop were conducted and the observational data were collected over a period of ten months. Pseudonyms have been used for districts, municipalities and the individual participants to ensure participant privacy.

### Inter-municipal services

In this section we present the different inter-municipal services included in the study. In addition to the services presented, the study comprises collaborative healthcare personnel and ICT support personnel in the municipalities.

The inter-municipal services included in the study were all services that had been newly established from one and a half to three months prior to the start of the study. The services were mainly organised as projects with full or partial external public funding.

In District 1, a dementia team, a substance abuse therapist and a psychologist were included. The dementia team consisted of one dementia coordinator, and one dementia contact in each municipality. Their work duties were primarily carried out in their respective municipality, where they held a part-time position on the dementia team. Their main task was to conduct dementia assessment among inhabitants with suspected dementia in the four municipalities of District 1. The substance abuse therapist and the psychologist had a full-time position in the inter-municipal services and served all four municipalities in the district. In addition, the project manager for the inter-municipal psychology and substance abuse services had a 20% inter-municipal position.

In District 2, the “palliation in Vik” project “[anonymised district designation]” had a project manager with a 30% inter-municipal position. The position had public funding: The project was established to enhance competence in palliation for district health personnel in the municipalities in the district. The occupational therapist had a full-time inter-municipal position and was supposed to serve as a resource in direct patient cases for all municipalities in the district. The Vik [anonymised district name] substance abuse team consisted of four positions, including a project manager. Their main task was to follow-up drug addicts in need of assistance in the municipalities in the district.

### Data collection

This case study examined communication and information sharing practices in inter-municipal healthcare services in two districts in Norway: District 1 and District 2. A total of 11 individual interviews were conducted, seven individual interviews in District 1 and four in District 2. In District 1, additional data were collected from one focus group, from two observational studies and from one workshop.

#### Interviews

In total, data from 11 individual interviews were collected based on information from healthcare managers, healthcare personnel, and ICT managers related to IMC. The interviews were conducted as semi-structured interviews lasting from 20 min to 2 h, and focused on communication and information sharing in inter-municipal healthcare services. Focus was specifically on how the inter-municipal work was conducted, how information was shared, what information was shared, and with whom information was shared, in addition to an identification of barriers related to communication and information sharing. The researcher made notes during the interviews. In addition, all interviews were audiotaped and transcribed verbatim.

#### Focus group interview

One focus-group interview was conducted with a dementia team, divided into two sessions of approximately one and one-half hours each with five participants. The interview was semi-structured and focused on the same topic as the individual interviews. One researcher made notes during the interview. In addition, all interviews were audiotaped and transcribed verbatim.

#### Workshop

In the workshop, seven participants from an inter-municipal team and a cooperating partner participated. The workshop focused on current work and the information process and identification of related barriers, and served as a verification of findings from interview and observation studies. This provided the basis for development of Business Process Modelling (BPM) [39, 40] to create externalised representation, including barriers and requirements of the processes of the inter-municipal dementia team. BPM was used as a basis for the development of a coordination tool for the inter-municipal cooperation. The researcher made notes during the workshop. In addition, it was audiotaped and transcribed, to ensure that no details were missed.

#### Observational studies

In addition, observational studies in three cases of the process of an inter-municipal work assignment were conducted. During field visits, researchers talked to healthcare personnel, observed actual operations, including sitting with healthcare personnel before, during and after a dementia assessment. The observations were done in the natural environment; including participants’ offices, the patients’ homes and at a meeting office at the health centre. Researchers also observed documentation practices in community nursing offices.

Elements of action research [41] appeared during the data collection. Questions during focus group interviews revealed different approaches between the municipalities. More importantly, the observation studies revealed unintended consequences based on the participants’ lack of awareness of each other’s work procedures. This resulted in changes in procedures, such as designing a routine for taking a phone call in addition to making an entry in the electronic health record (EHR). Changes in work processes based on findings from the observation studies were also reported.

### Analysis

The data from the transcribed interviews, observational studies and workshop were analysed. In total, there were 392 pages of text in Calibri font, size 12, space 1.5. An inductive approach inspired by qualitative content analysis, as described by Graneheim and Lundman [42] and recommended by Kohlbacher [43], was applied*.* The qualitative software tool Nvivo [44] was used for the analysis. Each individual interview, all observational notes and workshop notes were considered units of analysis. A thematic synthesis was used to identify, analyse and report themes across the different units. Thematic synthesis is one of the most common methods for synthesis of evidence in software engineering and has the advantage of providing a way to organise data from large and diverse sources [45, 46]. Each unit of analysis was first read through to obtain a sense of the whole. Then the text was coded and sorted into categories. The manifest content of the data was analysed. To answer RQ1, information sharing practices, we identified what information was shared, how and with whom. To answer RQ2, factors contributing to communication and information sharing, barriers were identified and coded and an inductive category development was compiled. During the analysis, the research group (the authors of this article) discussed findings to ensure an inter-disciplinary approach and understanding of the findings in the analysis-process. When all the units were analysed, categories were reviewed, merged and abstracted into main categories*.*

### Trustworthiness

To ensure credibility and the ability to elucidate the research question from a number of different perspectives, participants were chosen from various levels in the inter-municipal service and among different cooperative partners in the inter-municipal service. The participants were of various ages and both genders, and reflected perspectives on IMC from a broad workforce. The research team comprised persons with multi-disciplinary competence in ethics, eHealth, ICT and information systems. The complementary research background permits novel insights into the data and enhances confidence in the findings. The research team has had open discussions on the data’s consistency. Different perspectives were actively exploited in combination with various data collection methods to avoid unilateral focus, and thereby researcher bias.

## Results

Communication and information sharing practices were complex and characterised by multiple actors, information types, and channels for communication and information sharing. Findings suggested that the needs for communication and information sharing in IMC were not identified prior to establishment, neither by the involved municipalities nor the actors involved in the services. This resulted in several ad hoc solutions. The EHR was used actively for information sharing, but other communication channels like phone, face-to-face (FTF) and mails by post were often used as a substitute for, or alternative to, the EHR. Findings indicate that 1. IT capability and usability 2. Differences 3. Privacy, confidentiality and security and 4. Awareness are all factors that may enable barriers to communication and information sharing in inter-municipal healthcare services.

### Characteristics of information and communication practices in IMC

To answer research question 1 “What characterises the information and communication practices in inter-municipal healthcare services?” actors involved in communication and information sharing, information types, and how information is shared and communicated in inter- municipal healthcare services were identified.

#### Involved actors

Inter-municipal employees in healthcare services communicate and share information with multiple, different actors. In addition to communication amongst team members in the inter-municipal services, they communicate with employees in several municipal healthcare services, with social services, hospital services, and patients and relatives. See Table 2 for detailed results.

**Table 2:** Communication and information sharing actors

Actors	
Municipal healthcare services	• General Practitioner • Occupational therapist • Physical therapist • Habilitation services • Health clinics • Assistive technology services • Home care services • Intake services • Substance abuse and psychiatric services • Personal assistants • Psychologist
School, Police and Social services	• Child welfare • School • Labour and welfare services • Educational and Psychological Counselling Services • High school follow-up services • Correctional services
Hospital services	• Supervisors • Relevant departments
Patients/relatives	

#### Information types

Findings revealed two main categories of information: sensitive health information and non-sensitive information. See Table 3 for detailed results.

**Table 3:** Information types

Sensitive health information	Non-sensitive information
Declaration of consent	Coordinative information
Epicrisis	Information about inter-municipal service
Discuss patient cases	Team contact
Referral	Advice and guidance
Information on recommended actions, status after assessment and the tasks performed	Teaching
Assessment report	
Transfer note	
Report after patient-related meetings	

Related to patient treatment, different information needs to be shared before, during and after a direct patient contact. In addition, the inter-municipal employees had to communicate coordination tasks. Information about the inter-municipal services was shared. The IM-services possessed a specialised competence, and guiding, advising and training other municipal healthcare workers were often part of their work tasks. In addition, the need for informal communication between team members was highlighted by the participants.

#### Information methods

Finally, we identified how the information was shared and communicated in inter-municipal healthcare services. Findings revealed two main categories: Digital and non-digital communication, including several methods of information sharing. Specifically, findings implied that usually multiple actions were taken to provide information, and sensitive health information was often provided using a combination of both digital and non-digital methods. See Table 4 for detailed results.

**Table 4:** Information methods

Digital communication	Non-digital communication
Electronic Health Record (EHR)	Work book
E-mail	Work list
Web-pages	Fax machine
Local disc	Physical meetings
Video conference	Oral
	Paper-based
	Post
	Phone
	Print

### Factors that may enable barriers to communication and information sharing

In RQ2, findings from RQ1 are elaborated on:

“Which factors can contribute to barriers for communication and information sharing in newly established inter-municipal healthcare services?”

Findings indicate that there are many barriers to exchange of information. These barriers are related to usability and capability regarding the IT-systems, differences in both procedures and information systems, privacy, confidentiality and security issues and awareness amongst employees. These barriers may indicate that needs regarding communication and information sharing were not identified prior to the establishment of the IMC. On the contrary, the needs seem to have been met along the way.

#### IT capability and usability

The inter-municipal work was characterised by multiple sources for communication and information sharing. Findings suggested this was a result of IT capability and/or usability. Necessary functionality to support work and information flow in the EHR was either lacking or usability issues made it inaccessible. In particular, the lack of proper administrative and coordinative tools resulted in the need for multiple actions to provide correct information to the right person.

The tests performed by the dementia team were paper-based, but the results had to be reported in the EHR, leading to double actions as well as a risk of errors. The main collaborative municipal partners were the home care services and the GPs. The GPs had no system for achieving the test results digitally, so the test results had to be dispatched as paper-based documents by regular mail.

A crucial factor related to information sharing was the systems’ lack of efficiency and ease of use. The findings revealed a dissatisfaction with the EHR, among both those who recently implemented the system, and those who had had the system for a long period.

The personnel stated that the EHR lacked several functions. This resulted in a need for multiple alternative ways to document and share information. One example was that even though it was technically possible for the home care manager to read reports from the dementia team, she received no message saying that the report was accessible. The dementia coordinator had to make an additional phone call to inform that the report was completed and accessible, and that actions from the home care services should be taken.

Participant:

«…because no signs appears, and it is a new scheme and report [in the EHR], and that is inexpedient, it would have been nice if it blinked. In other words, one does need an additional communication channel to make sure there is something there. So, we found out that what works best for us right now is that he calls us, says this and that, and then I can read and provide information to the community nurse»

The systems in municipal homecare services were characterised by information overload, in addition to the multiple sources of communication and information sharing. In the home care services, a typical routine for the district nurse was first to check a printed list of her daily patient tasks. The list was updated with sticky notes and discontinued tasks were crossed out. She also had to check the “Black book” that provides additional patient information, such as «Get drugs at the pharmacy and Sarah is going to the doctor at 12 o’clock.» Then she checked the EHR which provided continuous information on all patients receiving service from the home care services. The home nurse stated that she did not bother reading entries from many days back, because she was provided with so much unnecessary patient information.

The personnel did not know about the technological solutions that could potentially make coordination easier. For example, no one knew if the calendar function to coordinate meeting times between municipalities worked. Technically, they had the possibility to access the information, but they did not know that they were expected to access it, nor how to do so, and this revealed that employees lacked sufficient training of the EHR and that the system was not intuitive in its design.

#### Differences

The communication and information sharing between the inter-municipal services and the municipal partners were characterised by different procedures based on available information technology.

In one district, some of the municipalities changed EHR vendors so that the municipalities in the district could benefit from using the same vendor. Nevertheless, even though the same vendor delivered the EHR, the EHR was adapted to the needs in each municipality. This led to different layout in the municipalities and a challenge for employees who met different content in the EHR in the various municipalities. Semantic translation between cooperative municipalities was also complicated because the various municipalities used different names for the same objects. In Norway, there are two equate but different written language variants. Complications were caused by the use of different written language variants in the municipalities within one district. The differences resulted in cumbersome practices for the inter-municipal employees, as they spent much time looking for the right place to document patient data.

In addition to differences in the EHR, findings also revealed differences in procedures. Although several persons might report a need for a dementia assessment using several different procedures, the GP always had to approve assessment done by the team. Our study found that this approval was not acquired in all assessments. The diversity in procedures for obtaining information laid down varying procedural requirements.

#### Privacy, confidentiality and security

The juridical issues concerning privacy, confidentiality and security were found to be a comprehensive area of concern. Specifically, access control and duty of secrecy were frequently discussed among the participants and new perspectives were highlighted and actualised due to the implementation of IMC. The ICT manager described the current access path to the EHR as both cumbersome and ineffective. The EHR was unsatisfactory in terms of differentiating necessary access; managers got very broad access to patient data, and a fictive/emergency username and password could be used during evening, nights and weekends if the nurses needed quick access to patient data when the manager was not present. All users could access patients registered in the system, even though the detailed information and journal notes were not accessible. The smaller municipalities provided wider access to patient information, compared to the larger municipalities.

The inter-municipal team members had their main employment in the municipal healthcare services, such as nursing homes or community nursing, and a part-time position in the inter-municipal service. Their access control was linked with their main position in the municipal healthcare services and was not adapted to needs regarding the inter-municipal service. Employees described a situation where there was a mismatch between how they were encouraged to deliver services and how security concerns complied with current legislation.

ICT-personnel stated:

“But I do hope that we can manage a solution which satisfy both privacy concerns and ensure accessibility. Because I do think that we must be able to provide a solution that makes patient safety and privacy concerns fit together instead of biting each other’s tails”

She continued:

“… It is a jungle and it is hard to identify which of the agencies is correct, and how to interpret today’s legislation and how it will be adapted. That is priority one. That’s where I think the national government has an extremely important job to do, in adapting the legislation to what they promote as good and expedient ways to work.”

The practitioners described practices where they tried to make the best of the situation with what they had available, even though the solutions they chose were not necessarily legal. Examples given included borrowing user names and passwords from colleagues to access the system or sending e-mails (outside the secure system) with patient initials. It was also expressed that denial of access to document patient information had implications for patient safety and quality of the service.

#### Awareness

Findings suggest that there was a lack of mutual awareness between employees in the inter-municipal services and their collaborators. The lack of awareness was related to employees’ whereabouts, when they were present, what information was received, how information was received and what was done with information received. In the small communities, employees often had several roles, and relevant patient information was shared randomly in arbitrary meeting arenas. This might indicate that in some cases, because the IMC was placed in small communities, information found its way despite, rather than because of, structured information flow. The established inter-municipal services were new in all the municipalities. They were largely implemented as isolated services with a lack of procedures for information sharing and integration with collaborating partners. There was a lack of awareness regarding work procedures between different stakeholders in the municipalities:

“the doorbell rings, and the daughter in law opens [the door]. It is the home care services that have come to administer the medication. They agree that she can come back later, when the dementia team have finished the assessment.” (field note)

Coordinative information between the inter-municipal dementia team and the home care services in the municipalities was found to be inadequate, resulting in interference in the assessment setting, the necessity to reschedule home care service routines and improper test conditions for the patient.

## Discussion

The inter-municipal work was characterised by multiple sources of communication and information sharing, and barriers to exchange of information were related to both usability and capability regarding the IT-systems, differences in both procedures and information systems, privacy, confidentiality and security issues and awareness amongst employees. Although the EHR is digital and information is supposed to be documented, it was found that communication and information sharing were conducted in several other ways outside the EHR. This indicates that the present functionality and use do not make it a sufficient tool for meeting the need for communication and information sharing in inter-municipal healthcare services. It is not known what employees expected prior to the establishment of the IMC. However, it is known that the employees got their experience during, rather than prior to, the implementation of the services. Findings revealed barriers concerning IT capability and usability-issues; the system lacked necessary functionality to perform work tasks and was cumbersome to use. The UTAUT-model defines effort expectancy as “the degree of ease associated with the use of the system” [33]. “Effort Expectancy” will, according to UTAUT, influence behavioural intentions, and eventually, Use Behaviour. Findings show that in addition to the use of digital communication and information sharing, non-digital methods were used extensively, either as a compliment to or instead of digital information sharing. Based on the UTAUT-model, both the negative experience regarding “Effort Expectancy” and “Facilitating conditions” will influence Behavioural intentions and Use Behaviour [33], and can be used to explain the extensive use of additional non-digital communication and information sharing methods.

In addition, the diversity amongst the collaborating municipalities negatively affected the use of EHR and was found to be a barrier to communication and information sharing. This was also the case related to judicial issues that prohibited access to necessary patient information across municipal borders. These barriers are related to a lack of an overarching facilitation of needs regarding communication and information sharing in inter-municipal services.

In recent decades there has been a shift away from information protection to information sharing across organisations [5]. Findings in the present study indicate that in the context of inter-municipal healthcare services, this is not the case. Findings suggest a tension between patient information privacy protection and the possibility of effective and qualitatively good services through IMC, requiring information sharing to facilitate continuity of care. Policy and information infrastructure do not enable sharing of sensitive patient information across municipal borders, but political reforms [36] promote this way of organizing health services. This creates barriers for employees in the services, as policy and practice represent opposites.

Differences were found to be a barrier to communication and information sharing. In the literature on inter-organisational integration and collaboration, one finds structural barriers such as different administrative boundaries, different laws and regulations, different information systems and databases [47, 48]. The present study indicates that this is also an important factor to be addressed in inter-municipal healthcare services, as it represents a barrier to communication and information sharing among the employees. The individual municipalities are structured bureaucratically and are shaped by local needs, resulting in a culture that treats information sharing in different ways. Differences is also related to barriers concerning lack of awareness. Awareness is described as “…an *understanding of the activities of others*, which provides a *context for your own activity*. This context is used to ensure that individual contributions are relevant to the groups activity as a whole,” [49] (p. 107). A lack of awareness was identified in terms of where employees were, when they were present, what information was received, how information was received and what was done with the information received. When inter-municipal employees work across different municipalities, much is required of them regarding knowledge of local procedures to be followed. The diversity amongst the municipalities challenges the possibility for employees to be aware of the locally adapted procedures regarding communication and information sharing; hence, this is specifically relevant to inter-municipal cooperation. When facilitating eHealth, infrastructure standards regarding interoperability, terminology and nomenclature are crucial [29], and support our findings that differences are factors that serve as barriers to information sharing.

Our study found barriers related to inter-organisational information sharing that were closely related to barriers regarding intra-organisational and intra-personal information sharing. As pointed out by Yang and Maxwell [5], the inter-organisational information sharing is interrelated with intra-organisational information sharing and intra-personal information sharing. Even though information was transferred from the inter-municipal service to the municipal service, there were inadequate routines in the municipalities to ensure that information was effectively and efficiently addressed. Intra-personal information sharing in various and random settings ensured necessary communication, and there was a need for multiple communication methods to ensure the information was received and handled properly. The traditional work processes among municipal actors had not been re-engineered due to the implementation of new inter-municipal services; as a consequence, information sharing routines were not in place. Our findings support the importance of addressing inter- and intra-organisational and intra-personal information sharing in inter-municipal healthcare services.

The complexity of the public sector has been widely addressed and has often been referred to as containing “wicked problems” [50–52]. Wicked problems are described to be unstructured, implying challenges regarding identification of causes and effects and therefore little consensus on the problem or solution [51]. Our findings identified a lack of preparation regarding information sharing needs in inter-municipal cooperation. Theory regarding wicked problems, nevertheless, implies that it is not possible to get a full view of the needs prior to establishment. This suggests that in addition to identifying information sharing needs in IMC, it is important to establish a strategy for how to manage challenges that arise along the way. Flexibility and the ability to adapt are regarded as positive traits of networks [16], and are a strategy for solving problems. They appear to have the potential to be a feasible strategy regarding information sharing in inter-municipal healthcare services. Information sharing is characterised by a continual development, due to the ongoing changes in the context, such as implementation of electronic messaging, the striving for commonalities in cooperating municipalities and so on. Findings suggest that the dilemma between the flexible nature of the IMC and the bureaucratic nature of the municipality creates challenges in anchoring the service. Changes and remedies can occur rapidly in the IMC, while the structures in the municipalities are inflexible and change takes more time.

### Practice and policy implications

Findings indicate a situation where there was a mismatch between how they were encouraged to deliver services and how security concerns complied with current legislation. In future reforms, national policymakers should make sure that recommendations pertaining to service delivery are matched with adapted legislation. Recently, there has been an increased focus on the importance of having managers facilitate information sharing and communication in networks [21, 22], but how and what areas managers should focus on have not been elucidated so far. Our study reveals a need for increased managerial focus on identifying needs regarding information sharing prior to establishment of new inter-municipal services. Employees experience that communication and information sharing across municipal borders is complex and involves several different actors, types of information and information sharing methods. Findings identified several factors experienced by employees that serve as barriers to communication and information sharing. To ensure satisfactory information sharing in newly established inter-municipal healthcare services, organisational, technical and policy factors must be considered prior to, and during, establishment and implementation. Findings in the present study indicate that an important area for managers to focus on, when establishing inter-municipal healthcare services, is the overarching facilitation of needs regarding communication and information sharing. This must include the needs of collaborating partners in the municipal healthcare services who must be aware of one other’s practices prior to establishment. The managers must also take into consideration the differences that are present in the involved municipalities. Information infrastructure should be adapted to new inter-municipal healthcare services and facilitate integration with existing municipal services. Managers must consider whether differences between the municipalities can be managed without too much effort on the part of the employees, or whether the individual municipalities should work toward a uniform practice to optimise inter-municipal services. Managers must also consider the juridical window of opportunity regarding information sharing across organisations. In the effort to identify important issues prior to establishment, it must be kept in mind that the complexity and wicked problems that may occur in inter-municipal healthcare services cannot necessarily be foreseen prior to establishment. Managers should include a strategy to solve problems as they arise. The flexible nature of the network organisation of IMC can promote this ongoing problem solving.

### Limitations

We acknowledge some limitations in our study. The geographical setting was limited and provided findings from two districts in Norway. It would be of great value to conduct similar studies in other districts in Norway, as well as in other countries. This could permit a comparison of whether the local context, such as statues and regulations, structures and cultural influences might affect the findings that have been revealed in the present study. The healthcare sector, as well ICT and jurisdiction, are experiencing rapid development. The time of the study might affect findings, as it will imply a change in those contextual factors. Furthermore, this study provides findings from the employee perspectives. Other perspectives, such as those of managers and policymakers can provide additional or contradictory findings and are necessary to obtain a complete view of information sharing in inter-municipal healthcare services. Further research is needed to address the limitations; whether new research confirms or refutes our findings, it will serve to bolster and expand needed knowledge in the field of inter- organisational information sharing.

## Conclusion

Communication and information sharing in newly established inter-municipal healthcare services are complex and characterised by several different collaborating actors, information sharing methods and information types. In the current study, organisational preconditions, technological limitations and policy issues paved the way for the experience of factors contributing to barriers regarding communication and information sharing in inter-municipal healthcare services. Factors like IT capability and usability, differences, privacy, confidentiality and security and awareness are contributing factors to the barriers experienced by the employees. Findings in the study support the need for a specific focus on the context of inter-municipal cooperation when assessing communication and information sharing. The perspective of the employees can provide useful insight, and findings may be of relevance for managers and policy makers in inter-municipal services. By focusing on the context of inter-municipal cooperation when assessing communication and information sharing in healthcare services, this article contributes to further theorizing on this particular field of research, and hence, closing the gap in existing knowledge.

## Abbreviations

BPM: Business Process Modelling
EHR: Electronic Health Record
FTF: Face to face
GP: General practitioner
ICT: Information and Communication Technology
IMC: Inter-municipal cooperation
IS: Information Systems
IT: Information technology
NSD: Norwegian Centre for Research Data
PACS: Picture Archiving and Communication Systems
PAS: Patient Administrative System
RIS: Radiology Information Systems
RQ: Research question
UTAUT: Unified Theory of Acceptance and Use of Technology

## References

[1] Valentijn PP, Schepman SM, Opheij W, Bruijnzeels MA (2013). Understanding integrated care: a comprehensive conceptual framework based on the integrative functions of primary care. Int J Integr Care.

[2] MacIntosh J, McCormack D (2001). Partnerships identified within primary health care literature. Int J Nurs Stud.

[3] Hulst R, Montfort A (2007). Inter-municipal cooperation: a widespread phenomenon. Inter-municipal cooperation in Europe.

[4] Gil-Garcia J. Ramon, Helbig Natalie, Ojo Adegboyega (2014). Being smart: Emerging technologies and innovation in the public sector. Government Information Quarterly.

[5] Yang T-M, Maxwell TA (2011). Information-sharing in public organizations: a literature review of interpersonal, intra-organizational and inter-organizational success factors. Gov Inf Q.

[6] Aanestad Margunn, Grisot Miria, Hanseth Ole, Vassilakopoulou Polyxeni (2017). Information Infrastructures within European Health Care.

[7] Holen-Rabbersvik E, Eikebrokk TR, Fensli RW, Thygesen E, Slettebo A (2013). Important challenges for coordination and inter-municipal cooperation in health care services: a Delphi study. BMC Health Serv Res.

[8] Thomson AM, Perry JL (2006). Collaboration processes: inside the black box. Public Adm Rev.

[9] Axelsson R, Axelsson SB (2006). Integration and collaboration in public health—a conceptual framework. Int J Health Plann Manag.

[10] Williamson OE. Markets and hierarchies, analysis and antitrust implications: a study in the economics of internal organization. New York: Free Press; 1975.

[11] Powell W (2003). Neither market nor hierarchy. Sociol Organ: Class Contemp Crit Read.

[12] Miles RE, Snow CC (1992). Causes of failure in network organizations. Calif Manag Rev.

[13] Miles RE, Snow CC (1986). Organizations: new concepts for new forms. Calif Manag Rev.

[14] Powell W (1990). Neither market nor hierarchy: network forms of organization. Res Organ Behav.

[15] Jones C, Hesterly WS, Borgatti SP (1997). A general theory of network governance: exchange conditions and social mechanisms. Acad Manag Rev.

[16] Provan KG, Kenis P (2008). Modes of network governance: structure, management, and effectiveness. J Public Adm Res Theory.

[17] Huang K, Provan KG (2007). Structural embeddedness and organizational social outcomes in a centrally governed mental health services network. Public Manag Rev.

[18] O’Toole LJ, Meier KJ (2004). Public management in intergovernmental networks: matching structural networks and managerial networking. J Public Adm Res Theory.

[19] Kort M, Klijn EH (2011). Public–private partnerships in urban regeneration projects: organizational form or managerial capacity?. Public Adm Rev.

[20] Kickert WJ, Klijn E-H, Koppenjan JF. Managing complex networks: strategies for the public sector. London: Sage; 1997.

[21] Koliba C, Wiltshire S, Scheinert S, Turner D, Zia A, Campbell E (2017). The critical role of information sharing to the value proposition of a food systems network. Public Manag Rev.

[22] Vangen S (2017). Developing practice-oriented theory on collaboration: a paradox lens. Public Adm Rev.

[23] Jewett T, Kling R (1991). The dynamics of computerization in a social science research team: a case study of infrastructure, strategies, and skills. Soc Sci Comput Rev.

[24] Star SL, Ruhleder K. Steps towards an ecology of infrastructure: complex problems in design and access for large-scale collaborative systems. North Carolina: In: Proceedings of the 1994 ACM conference on computer supported cooperative work. 1994. p. 253–64.

[25] Bowker Geoffrey C., Baker Karen, Millerand Florence, Ribes David (2009). Toward Information Infrastructure Studies: Ways of Knowing in a Networked Environment. International Handbook of Internet Research.

[26] EHEALTH. [https://ec.europa.eu/health/ehealth/policy_en]. Accessed 1 Dec 2017.

[27] Commission of the European Communities. e-Health – making healthcare better for European citizens: An action plan for a European e-Health Area. 2004. https://ec.europa.eu/digital-single-market/en/news/e-health-making-healthcare-better-european-citizens-action-plan-european-e-health-area. Accessed 1 Dec 2017.

[28] European Commission. eHealth Action Plan 2012-2020 – innovative healthcare for the 21st Century. Communication from the Commission of the European Parliament, The Council, The European Economic and Social Committee, and the Committee of the Regions; Brussels; 2012. http://ec.europa.eu/information_society/newsroom/cf/dae/document.cfm?doc_id=4188. Accessed 1 Dec 2017.

[29] Aanestad M, Grisot M, Hanseth O, Vassilakopoulou P, Aanestad M, Grisot M, Hanseth O, Vassilakopoulou P (2017). Information infrastructures for eHealth. Information Infrastructures within European Health Care.

[30] International Organization for Standardization (2005). ISO/TR 20514 2005. Health informatics — Electronic health record — Definition, scope and context.

[31] Winter Alfred, Haux Reinhold, Ammenwerth Elske, Brigl Birgit, Hellrung Nils, Jahn Franziska (2010). Health Information Systems. Health Information Systems.

[32] Gil-Garcia JR, Chengalur-Smith I, Duchessi P (2007). Collaborative e-Government: impediments and benefits of information-sharing projects in the public sector. Eur J Inf Syst.

[33] Venkatesh V, Morris MG, Davis GB, Davis FD (2003). User acceptance of information technology: toward a unified view. MIS Q.

[34] Hennington A, Janz BD (2007). Information systems and healthcare XVI: physician adoption of electronic medical records: applying the UTAUT model in a healthcare context. Commun Assoc Inf Syst.

[35] Williams MD, Rana NP, Dwivedi YK (2015). The unified theory of acceptance and use of technology (UTAUT): a literature review. J Enterp Inf Manag.

[36] Report No. 47 (2008–2009) to the Storting (2009). The coordination reform, proper treatment –at the right place and right time.

[37] Yin RK. Case study research: design and methods. Thousand Oaks, CA: Sage publications; 2013.

[38] Patton MQ (1999). Enhancing the quality and credibility of qualitative analysis. Health Serv Res.

[39] Reichert Manfred (2011). What BPM Technology Can Do for Healthcare Process Support. Artificial Intelligence in Medicine.

[40] Emanuele J, Koetter L (2007). Workflow opportunities and challenges in healthcare. 2007 BPM & Workflow Handbook.

[41] Reason P, Bradbury H. Handbook of action research: participative inquiry and practice. Thousand Oaks: Sage; 2001.

[42] Graneheim UH, Lundman B (2004). Qualitative content analysis in nursing research: concepts, procedures and measures to achieve trustworthiness. Nurse Educ Today.

[43] Kohlbacher F (2006). The use of qualitative content analysis in case study research.

[44] QSR International Pty Ltd. (2012). NVivo qualitative data analysis software.

[45] Cruzes DS, Dybå T (2011). Research synthesis in software engineering: a tertiary study. Inf Softw Technol.

[46] Pope C, Mays N, Popay J (2007). Synthesizing qualitative and quantitative health evidence.

[47] Van Raak A, Mur-Veeman I, Paulus A (1999). Understanding the feasibility of integrated care: a rival viewpoint on the influence of actions and the institutional context. Int J Health Plann Manag.

[48] van Raak A. Integrated care in Europe: description and comparison of integrated care in six EU countries. Maarssen: Elsevier gezondheidszorg; 2003.

[49] Dourish P, Bellotti V. Awareness and coordination in shared workspaces. In: Proceedings of the 1992 ACM conference on computer-supported cooperative work. New York: ACM. 1992. p. 107–14.

[50] Ferlie E, Fitzgerald L, McGivern G, Dopson S, Bennett C (2011). Public policy networks and ‘wicked problems’: a nascent solution?. Public Adm.

[51] Roberts N (2000). Wicked problems and network approaches to resolution. Int Public Manag Rev.

[52] Horn RE, Weber RP (2007). New tools for resolving wicked problems: Mess mapping and resolution mapping processes.

[53] Association WM. World Medical Association Declaration of Helsinki: ethical principles for medical research involving human subjects. Jama. 2013;310(20):2191. 10.1001/jama.2013.281053 24141714

